# Molecular characterization of *Escherichia coli* virulence markers in neonatal and postweaning piglets from major pig-producing districts of Uganda

**DOI:** 10.1186/s12917-024-04092-x

**Published:** 2024-05-27

**Authors:** Margaret Tusiime, Frank. N. Mwiine, Mathias Afayoa, Steven Arojjo, Joseph Erume

**Affiliations:** 1 Department of Biosecurity, Ecosystems and Veterinary Public Health, College of Veterinary Medicine, Animal Resource and Biosecurity, Makerere University, Kampala, Uganda; 2 Department of Biomolecular Resources and Biolab Sciences, College of Veterinary Medicine, Animal Resource and Biosecurity, Makerere University, Kampala, Uganda; 3 Department of Veterinary Pharmacy, Clinical and Comparative Medicine, College of Veterinary Medicine, Animal Resource and Biosecurity, Makerere University, Kampala, Uganda; 4 Department of Sociology and Anthropology, College of Humanities and Social Sciences, Makerere University, Kampala, Uganda; 5 Department of Biotechnical and Diagnostic Sciences, College of Veterinary Medicine, Animal Resource and Biosecurity, Makerere University, Kampala, Uganda

## Abstract

**Background:**

Piggery production is highly constrained by diseases, with diarrhoea in piglets being a major cause of economic losses to smallholder farmers in Uganda. Enterotoxigenic *Escherichia coli* (ETEC) is thought to be one of the major etiologies of this diarrhoea. A cross-sectional study was carried out in two high pig-producing districts of Uganda with the aim of determining the significance of piglet diarrhoea and the pathogenic determinants of causative *E. coli*.

**Methodology:**

A total of 40 households with piglets were visited in each district for a questionnaire survey and faecal sample collection. The questionnaire-based data collected included; demographic data and pig management practices. *E. coli* were isolated from diarrheic (43) and non-diarrheic (172) piglets and were subjected to antimicrobial susceptibility testing against nine commonly used antimicrobial agents. The *E. coli* isolates were further screened for the presence of 11 enterotoxin and fimbrial virulence gene markers using multiplex polymerase chain reaction. Data entry, cleaning, verification and descriptive statistics were performed using Microsoft Excel. Statistical analysis to determine any association between the presence of virulence markers and diarrhea in piglets was done using SPSS software (Version 23), with a *p* value of less than 0.05 taken as a statistically significant association.

**Results:**

*Escherichia coli* were recovered from 81.4% (175/215) of the faecal samples. All the isolates were resistant to erythromycin, and most showed high resistance to tetracycline (71%), ampicillin (49%), and trimethoprim sulfamethoxazole (45%). More than half of the isolates (58.3%) carried at least one of the 11 virulence gene markers tested. EAST1 was the most prevalent virulence marker detected (35.4%), followed by STb (14.8%). Expression of more than one virulence gene marker was observed in 6.2% of the isolates, with the EAST1/STa combination being the most prevalent. Three adhesins; F17 (0.6%), F18 (6.3%) and AIDA-I (0.6%) were detected, with F18 being the most encountered. There was a statistically significant association between the occurrence of piglet diarrhoea and the presence of the AIDA-1 (*p value* = 0.037) or EAST1 (*p value* = 0.011) gene marker among the isolates.

**Conclusion and recommendation:**

The level of antimicrobial resistance among *E. coli* isolates expressing virulence markers were high in the sampled districts. The study established a significant association between presence of EAST1 and AIDA-I virulence markers and piglet diarrhea. Further studies should be carried out to elucidate the main adhesins borne by these organisms in Uganda and the actual role played by EAST1 in the pathogenesis of the infection since most isolates expressed this gene.

**Supplementary Information:**

The online version contains supplementary material available at 10.1186/s12917-024-04092-x.

## Introduction

Pig production is a very important global economic activity that is driven by increased demand for animal source proteins resulting from exponential human population growth [1, 2]. The relatively limited investment required in the local piggery enterprise coupled with the fast maturing and high prolificacy of the pigs make the enterprise ideal for most poor rural communities [2, 3] The sector is, however, constrained by several factors, key among which are diseases that highly affect piglets [4]. These diseases affect the survivability and performance of piglets, which in turn negatively affects enterprises that are highly dependent on the performance of piglets [5, 6]. One of the major clinical conditions in piglets is diarrhoea, which can be a result of several factors; however, *Escherichia coli* has been highly implicated in the diarrhoea observed in piglets [7, 8].

*Escherichia coli-*induced diarrhoea, colibacillosis, is primarily caused by enterotoxigenic *Escherichia coli* pathotypes [8]. Worldwide, over two different diarrhoea syndromes are recognized: neonatal diarrhoea in piglets from 1 to 7 days of age and postweaning diarrhoea, which manifests approximately one week following weaning [9]. ETEC associated with piglet diarrhoea expresses one or more fimbrial adhesins (F4, F5, F6, F18 and F41) along with enterotoxins STa, STb, LT or their combinations [4]. In addition to the latter, a number of other adhesins have also been reported among these strains, including F42, F165 and AIDA-I [10]. The Uganda piggery system experiences more than 30% preweaning piglet mortality, most of which is attributed to infectious diarrhoeal diseases [11]. This has prompted research to generate evidence of the role of *E. coli* as an etiology of piglet diarrhoea/mortalities in Uganda. Preliminary research identified ETEC expressing enterotoxin genes (STa and STb) and F4 adhesins in preweaning piglets in Uganda [1]. The purpose of this study was to expand this effort to the major pig-producing districts of Uganda with the aim of elucidating virulence markers, husbandry practices in small-scale piggeries and antimicrobial resistance levels of these organisms toward sustainable mitigation of colibacillosis losses.

## Materials and methods

### Study area and study population

The study targeted smallholder pig producers in two of the major pig-producing districts [12] of Uganda that is Masaka (0.4464 S, 31.9018 E) and Mukono (0.3549 N, 32.7520 E) districts located in central Uganda, Buganda subregion. These two districts are among the four high pig producing districts with a contribution to the pig population of 40.2% in central region and 16.5% in the whole country [13]. The study was performed in four sub counties in each of the selected districts. The study group was piglets under two months of age (new-borns and weaners) from selected farms.

### Study design

This was a cross-sectional study carried out from December 2021 to October 2022 involving the characterization of virulence markers and assessment of the antimicrobial susceptibility of *E. coli* to selected drugs. The study entailed a questionnaire survey to determine the husbandry practices under which the piglets were raised and faecal sample collection from the animals. Visits were made to the Veterinary Offices in the study districts to introduce the project, and with the help of the District Veterinary Officers (DVOs), four Sub Counties in each district were selected to contribute animals for study. Subsequently, ten households/pig farmers were selected for study from each of the sub counties. These farmers were identified by the snowballing method [14] with the help of field guides (paraprofessionals and veterinarians) assigned by the DVOs.

### Sample size determination

The sample size (n) for this study was determined based on an established formula (Thrusfield, 2005). Based on the findings of a previous study by Kallau et al. [15], the expected prevalence of *E. coli* in the faecal matter of pigs was 85.4% [15].

$$n=\frac{{Z}^{2}p(1-p)}{{d}^{2}}$$

where n is the sample size, *Z* is the statistical value at the 95% confidence interval, *d* is the absolute precision level with a value of 5% and *P* is the prevalence of *Escherichia coli*. With *Z* = 1.96, *P* = 0.854 and *e* = 5%, this yields *n* = 192 faecal samples. This was rounded to 200 samples, which is approximately 100 faecal samples per district.

### Ethical approval

This study entailed questionnaire administration to the pig farmers as well as fecal sampling from piglets. Therefore, the study procedure was assessed and approved by the School of Biosecurity, Biotechnical and Laboratory Sciences (SBLS), College of Veterinary Medicine, Animal Resources and Biosecurity (COVAB), Makerere University, Uganda on the 11th November 2021, reference number (SBLS.JE.2021) and by the Uganda National Council of Science and Technology (UNCST), approval number A190ES.

### Field data collection

#### Questionnaire survey

A pig herd level closed and semi closed questionnaire tool was developed for this study (Supplementary file_Questionnaire_English) consisting of 41 questions was administered to 80 household heads from whose piglets’ faecal samples were obtained. The data obtained included demographic information, pig management and husbandry practices, and occurrence of piglet diarrhoea, among other data. With the help of field guides, 10 households with piglets were selected in each of the sub counties, and a questionnaire was administered. In total, 40 questionnaires were administered per district.

#### Faecal sample collection

Rectal swabs from two piglets, identified as either diarrhoeic or non-diarrhoeic, from each litter were obtained as aseptically as possible. Each swab was placed in a sterile Falcon tube containing sterile peptone water as the transport medium. The swabs were transported under cold chain in a cool box with ice packs to Makerere University microbiology Laboratory at the College of Veterinary Medicine, Animal Resources and Biosecurity (CoVAB), Makerere University for analysis. The samples were temporally stored at 4 °C until further processing and analysis.

### Isolation and identification of *E. Coli*

Standard bacteriological procedures were followed for the isolation of *E. coli*. Briefly, faecal samples were enriched overnight in sterilized buffered peptone water at 37 °C, after which the samples were inoculated onto MacConkey agar. The suspected *E. coli* colonies were then identified using a combination of the following: colony characteristics, different standard biochemical tests such as the indole test, methyl red test, Voges Proskauer test and citrate test (IMViC tests) and typical morphology after Gram staining. The preservation of the isolates was performed using glycerol and brain heart infusion broth. The process involved suspending a pure colony of *E. coli* in 700 µl of brain heart infusion broth and culturing it overnight. This was then added to 30% (final concentration) sterilized glycerol in 1 ml cryovials. The mixture was vortexed to allow for uniform mixing and then preserved at -20 °C awaiting antimicrobial susceptibility testing and molecular characterization for the virulence markers.

### Antimicrobial susceptibility profiles of *E. Coli* isolates

Antibiograms of all the isolates were determined using the Kirby-Bauer disc diffusion technique [16] employing Muller Hinton Agar. A total of nine antimicrobials, including nalidixic acid (NA), trimethoprim sulfamethoxazole (SxT), gentamicin (CN), chloramphenicol (C), ciprofloxacin (CIP), erythromycin (E), kanamycin (K), ampicillin (AM), and tetracycline (Te), were tested. The drug inhibition zones were measured, and the results were interpreted as resistant (R), intermediate (I) or susceptible (S) according to the Clinical and Laboratory Standards Institute guidelines, 2018 [17].

### Molecular characterization of *E. coli* virulence markers

#### *E. coli* DNA extraction

The boiling method was used to extract DNA [18]. Briefly, 50 µl of the preserved isolate was placed in a 1.5 ml Eppendorf tube and centrifuged at 4000 rpm for 10 min. The supernatant was decanted, and 150 µl of 1x phosphate-buffered saline (PBS) solution was added to the pellet and vortexed. The mixture was then boiled in a water bath set at 70 °C for 10 min to lyse the cells and then cooled at -20 °C. The cooled mixture was centrifuged at 13,000 rpm for 5 min at 4 °C, and then the supernatant containing genomic DNA was aliquoted into a new tube and stored at -20 °C pending PCR amplification.

#### Detection of virulence markers using multiplex PCR

The genes targeted included 7 adhesins (F4, F5, F6, F17, F18, F41, AIDA-1) and 4 *E. coli* enterotoxins (STa, STb, LT and EAST1). All these virulence markers were detected by multiplex PCR using specific primers as presented in Table 1. The sizes of the target amplicons for the different primers are reflected in Table 1. PCR amplification was performed using One Taq DNA polymerase (BioLabs) in a reaction volume of 12.5 µl, comprising 0.25 µl of the forward and reverse primers, 6.25 µl of One Taq 2X Master Mix, 1.25 µl of nuclease-free water and 2 µl of the DNA template. PCR conditions comprised an initial denaturation at 94 °C for 30 s, followed by 35 cycles of denaturation at 94 °C for 30 s, annealing at 55 °C for 1 min, and extension at 68 °C for 1 min and thereafter a final extension at 68 °C for 5 min [19]). DNA from the in-house *E. coli* strains 853/67; O149 (F4^+^, F6^+^, LT^+^, STa^+^, STb^+^ and EAST1^+^), and Bd 60/84 I; O141(F18^+^, VT2e^+^, STa^+^ (NVI, Uppsala, Sweden) and a blank sample without DNA were used as positive and negative controls, respectively.

**Table 1:** Primers used in the study

No.	Oligo Name	Sequence (5̍->3̍)		Base Pair size (bp)		Reference		
1	LT FOR LT REV	ATTTACGGCGTTACTATCCTC TTTTGGTCTCGGTCAGATATG		280		[20]		
2	STa FOR	TCTTTCCCCTCTTTTAGTCAG		166		[21]		
	STa REV	ACAGGCAGGATTACAACAAAG						
3	STb FOR	GCCTATGCATCTACACAATC		279		[21]		
	STb REV	TGAGAAATGGAXAATGTCCG						
4	EAST1 FOR	CCATAACACAGTATATCCGA		111		[22]		
	EAST1 REV	GGTCGCGAGTGACGGCTTTGT						
5	F4 FOR F4 REV	GGTCGCGAGTGACGGCTTTGT CCACTGAGTGCTGGTAGTTACAGCC		792		[23]		
6	F5 FOR F5 REV	TGCGACTACCAATGCTTCTG TATCCACCATTAGACGGAGC		450		[24]		
7	F6 FOR F6 REV	TCTGCTCTTAAAGCTACTGG AACTCCACCGTTTGTATCAG		333		[25]		
8	F17 FOR F17 REV	GGGCTGACAGAGGAGGTGGGGC CCCGGCGACAACTTCATCACCGG		411		[26]		
9	F18 FOR F18 REV	GTGAAAAGACTAGTGTTTATTTC CTTGTAAGTAACCGCGTAAGC		510		[24]		
10	F41 FOR F41 REV	GAGGGACTTTCATCTTTTAG AGTCCATTCCATTTATAGGC		431		[24]		
11	AIDA-I FOR AIDA-I REV	TGCAAACATTAAGGGCTCG CCGGAAACATTGACCATACC		450		[27]		

### Agar gel electrophoresis

The PCR amplicons (4 µl) and DNA ladder were loaded and electrophoresed on a 2% (w/v) agar rose gel in 1x TAE buffer containing ethidium bromide (1.5 µg/ml) at 125 V for 45 min. Electrophoresed amplicons were visualized using ultraviolet transillumination and documented using an ENDURO GDS documentation system (Labnet International, USA, New Jersey).

### Data analysis

The data obtained were entered into Excel spreadsheets, where they were verified and cleaned. The data were then exported and analyzed statistically using SPSS software (Version 23). Descriptive, graphical, and summary statistics were performed using Microsoft Excel. A chi-square test was used to determine any association between the presence of virulence markers and diarrhoea in piglets, with a *p value* of less than 0.05 taken as a statistically significant association.

## Results

### Sociodemographic characteristics of the household heads

Eighty households with piglets of target ages were interviewed. Most of the respondents (75%) were males, and the majority (92.5%) had attained formal education. Most of the respondents (73.8%) were above the age of 40 years (Table 2). When the demographics of the two study districts were compared, Masaka had more respondents who had attained formal education than Mukono, and this difference was statistically significant (*p value* = 0.001). In both districts, most households owned up to 10 pigs, with exotic breeds being mainly kept (Table 3).

**Table 2:** Characteristics of household heads

Variable	Category	Frequency (%)
Head of the household	Female	20 (25)
	Male	60 (75)
Level of Education	None	6 (7.5)
	Primary	26 (32.5)
	Secondary	24 (30)
	Tertiary	24 (30)
Age of the head in years	21–30	5 (6.3)
	31–40	16 (20)
	41–50	20 (25)
	51–60	22 (27.5)
	> 61	17 (21.3)
Religion of the household head	Born again Christian	9 (11.3)
	Catholic	41 (51.2)
	Protestant	30 (37.5)
Occupation of the Household head	Peasant/farmer	43 (53.8)
	Civil Servant	20 (25)
	Self employed	10 (12.5)
	Other	7 (8.8)

**Table 3:** Comparing the social demographics for the two districts

Variable		Mukono *n*, (%)	Masaka *n*, (%)	*p* value
Sex	Female	7(17.5)	13(32.5)	0.196
	Male	33(82.5)	27(67.5)	
Level of education	None	5(12.5)	1(2.5)	0.001
	Primary	4(10)	21(52.5)	
	Secondary	17(42.5)	7(17.5)	
	Tertiary	13(32.5)	11(27.5)	
Age of family Head	21–30	3(7.5)	2(5)	0.256
	31–40	9(22.5)	7(17.5)	
	41–50	13(32.5)	7(17.5)	
	51–60	7 (17.5)	15 (37.5)	
	> 61	8(20)	9(22.5)	
Religion	Born again	9(22.5)	0(0.0)	< 0.001
	Catholic	8(20)	33(82.5)	
	Protestants	23(57.5)	7(17.5)	
Occupation	Civil servant	12(30)	8(20)	0.064
	Peasant	22(55)	20(50)	
	Self employed	5(12.5)	5(12.5)	
	Others	0(0)	7(17.5)	
Breed of pigs	Local	12(30)	13(32.5)	0.887
	Exotic	15(37.5)	16(40)	
	Both	13(32.5)	12(27.5)	
Size of pig herd	1 to 10	20(50)	22(55)	0.373
	11 to 20	12(30)	7(17.5)	
	21–30	5(12.5)	4(10)	
	> 31	3(7.5)	7(12.5)	

### Isolation and identification of *E. Coli* from faecal samples

The overall prevalence of *E. coli* from faecal samples from piglets for the two districts (Masaka and Mukono) was 81.4% (175/215), as shown in Table 4. There were 43 (20%) piglets presenting with signs of diarrhoea, 32 (74.4%) of which were confirmed to have *E. coli*, while 172 samples were from piglets without typical signs of diarrhoea, 83.1% of which possessed the *E. coli* bacterium.

**Table 4:** Prevalence of *E. coli* in diarrhoeic and non-diarrhoeic piglet faecal matter in Mukono and Masaka districts

District	E. coli		Total
	No %, *n*	Yes %, *n*	%, *n*
Masaka	20.4 (22)	79.6 (86)	50.2 (108)
Mukono	16.8 (18)	83.2 (89)	49.8 (107)
Total	18.6 (40)	81.4 (175)	100 (215)
**Diarrhoea Status**			
Negative	16.9 (29)	83.1 (143)	80 (172)
Positive	25.6 (11)	74.4 (32)	20 (43)
Total	18.6 (40)	81.4(175)	100 (215)

### Antimicrobial susceptibility profiles of *E. Coli* isolates

**Fig. 1:**
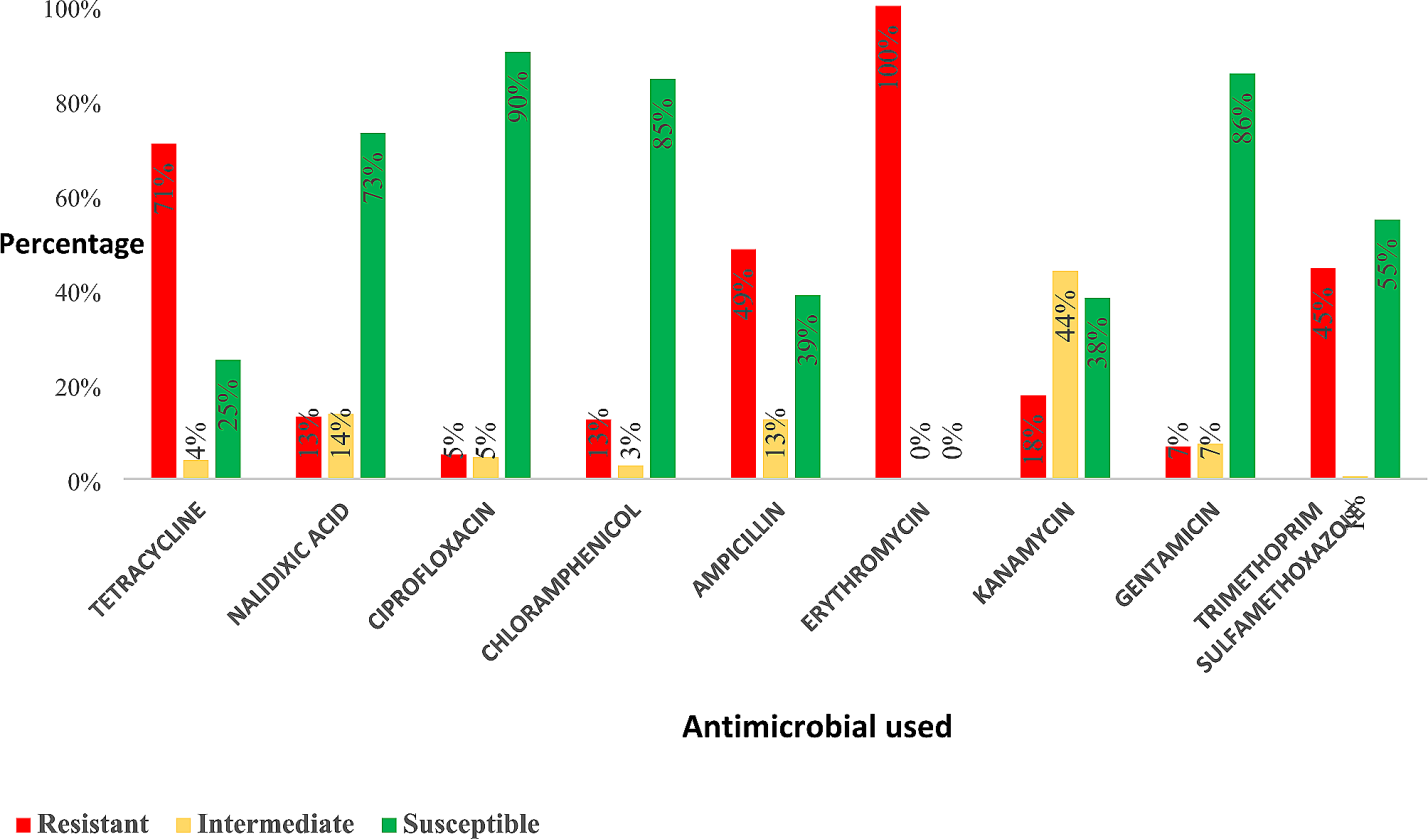
Shows the overall antimicrobial susceptibility of isolates from the two districts

**Fig. 2:**
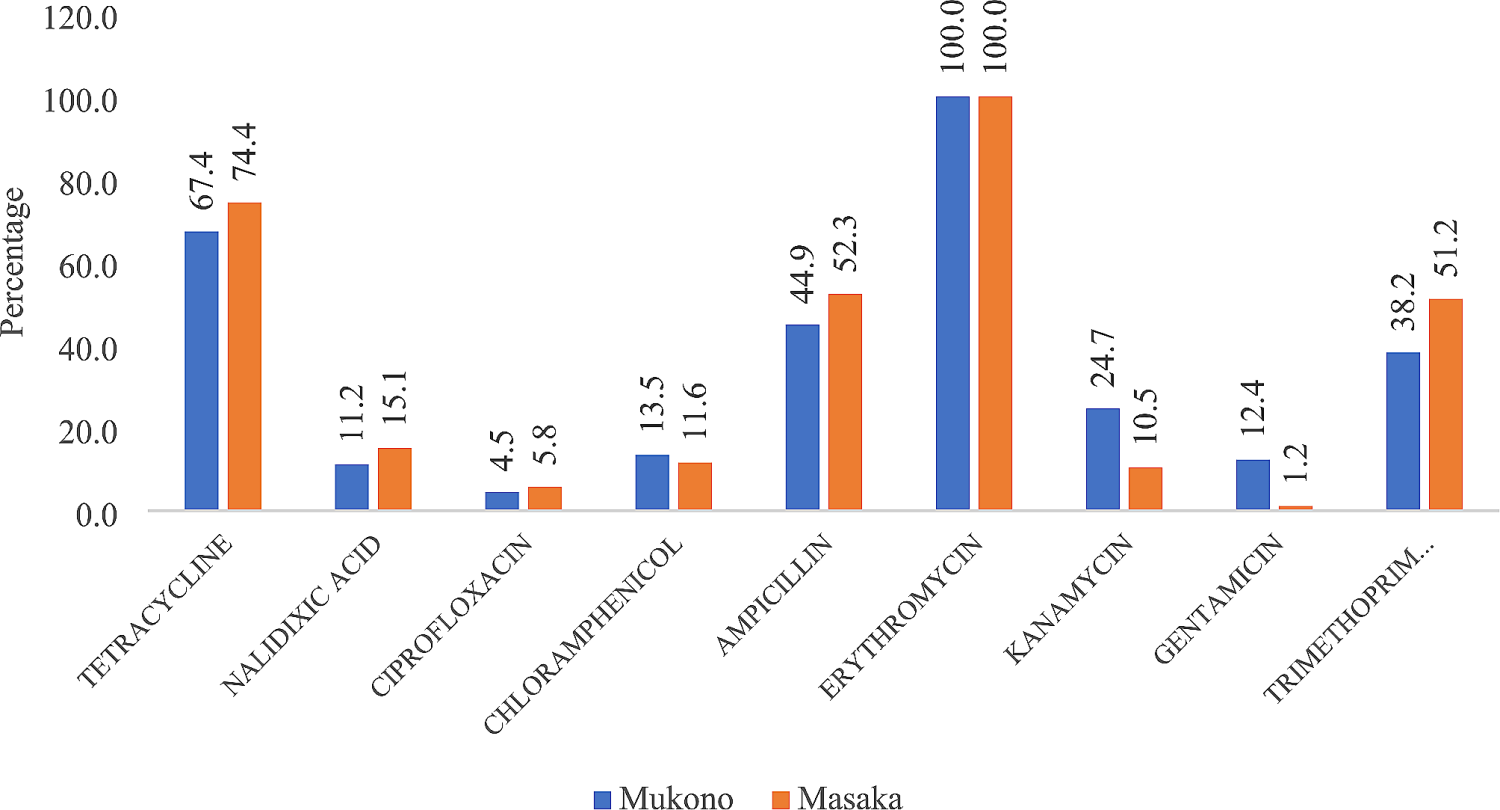
Resistance level against specific antimicrobials by district

The overall picture of the drug susceptibility of the 175 isolates from the two districts is depicted in Fig. 1. All the isolates were found to be resistant to erythromycin, and 71% showed resistance to tetracycline. There was high susceptibility to drugs such as nalidixic acid (73%), chloramphenicol (85%), ciprofloxacin (90%), and gentamicin (86%). Comparison of the specific antimicrobial resistance of the isolates from the two districts revealed a similar trend in both districts (Fig. 2). Interestingly, multidrug resistance (resistance to three or more drug classes) was high (*n* = 98, 56%).

### Molecular characterization of virulence markers of *E. Coli* isolates

#### Prevalence of virulence gene markers

**Fig. 3:**
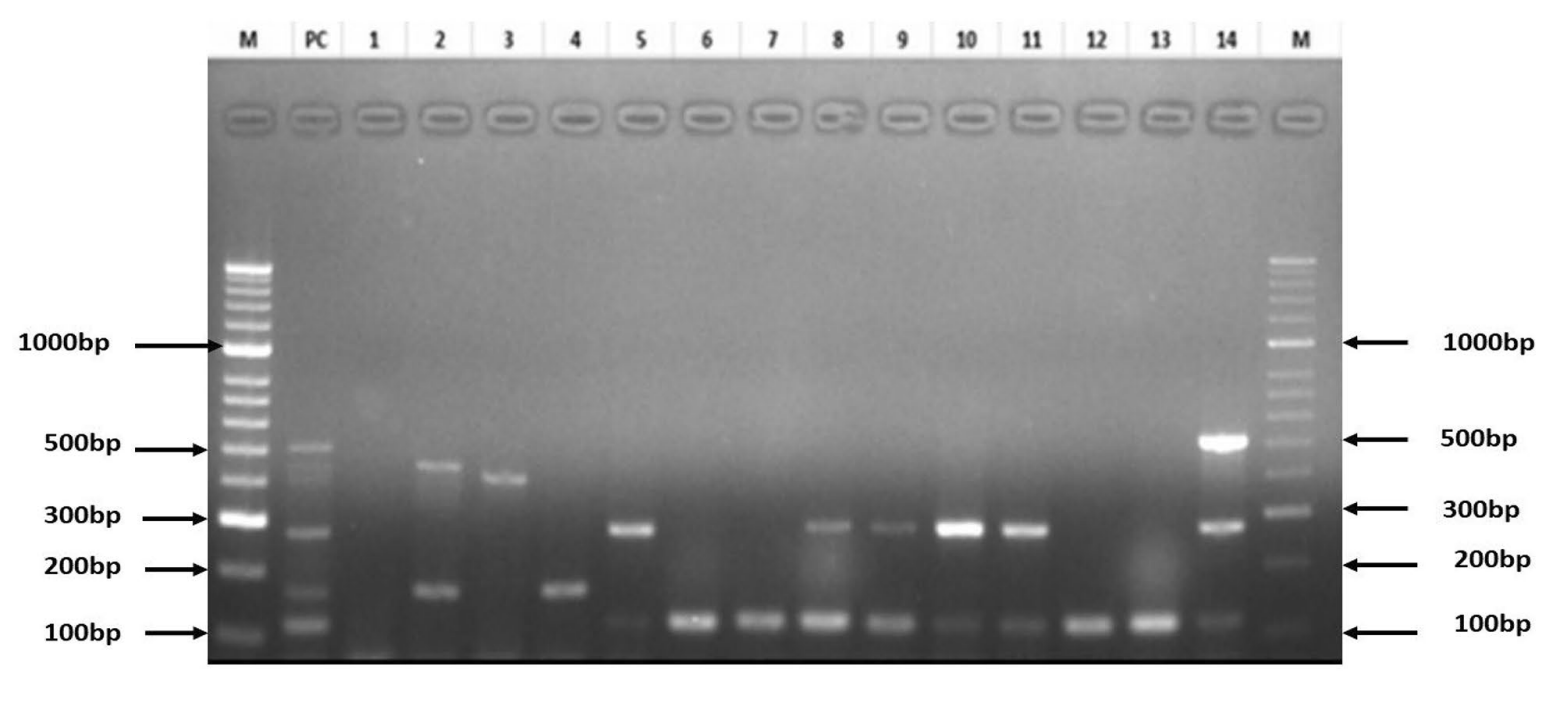
Representative gel of the PCR detection of virulence markers. Lanes M- 100 bp ladder, PC- positive control, 1- negative control, Lanes 2 to 14 are the test *E. coli* isolates screened. The amplicons were run on a 2% agarose gel and detected by ultraviolet transillumination and documented

Out of the 175 isolates, 102 (58.3%) carried at least one of the *E. coli* virulence markers screened. A few (6.3%) of the isolates carried adhesin markers (F17, AIDA-I and F18). A total of 21.7% had heat-stable enterotoxin markers (STa and STb), whereas the EAST1 gene marker was detected in 35.4% of the isolates, as shown in Table 5. A representative gel is shown in Fig. 3. Several (17.7%) isolates expressed more than one virulence marker, with the EAST1 and STb combination being the most frequently expressed (Table 6).

**Table 5:** Proportions of the *E. coli* virulence markers tested

Virulence Marker, *n* (%)	Masaka, *n* (%)	Mukono, *n* (%)	Total, *n* (%)
LT	0 (0)	0 (0)	0 (0)
STa	8 (9.3)	4 (4.5)	12 (6.9)
STb	15 (17.4)	11 (12.4)	26 (14.8)
EAST1	33 (38.4)	29 (32.6)	62 (35.4)
F4	0 (0)	0 (0)	0 (0)
F5	0 (0)	0 (0)	0 (0)
F6	0 (0)	0 (0)	0 (0)
F17	1 (1.2)	0 (0)	1 (0.6)
F41	0 (0)	0 (0)	0 (0)
AIDA-I	1 (1.2)	0 (0)	1 (0.6)
F18	1 (1.2)	10 (11.2)	11 (6.3)

**Table 6:** Occurrence of *E. coli* expressing multiple virulence markers in Masaka and Mukono districts

Virulence Marker combination, *n* (%)	Masaka, *n* (%)	Mukono, *n* (%)	Total, *n* (%)
EAST1/STb	12 (14.0)	5 (5.6)	17 (9.7)
EAST1/F18	0 (0)	2 (2.2)	2 (1.1)
EAST1/STa	2 (2.3)	1 (1.1)	3 (1.7)
STa/STb	0 (0)	1 (1.1)	1 (0.6)
STb/F18	0 (0)	2 (2.2)	2 (1.1)
STa/STb/F18	0 (0)	1 (1.1)	1 (0.6)
STa/F18/EAST1	0 (0)	1 (1.1)	1 (0.6)
F17/EAST1/STa	1 (1.2)	0 (0)	1 (0.6)
EAST1/STa/STb	3 (3.5)	0 (0)	3 (1.7)
AIDA-1/EAST1	1 (1.2)	0 (0)	1 (0.6)
F18/STb/EAST1	1 (1.2)	0 (0)	1 (0.6)

#### Association of *E. Coli* virulence markers with diarrhoea status of the sampled piglets

There was a significant association between the presence of the EAST1 gene in the *E. coli* isolates and the occurrence of diarrhea in the sampled piglets (*p value* of 0.011). Similarly, the presence of the AIDA-I gene was significantly associated with diarrhea (*p value* of 0.037). However, there was no significant association between the presence of F17, STa, STb and F18 and the occurrence of diarrhoea in piglets from the Mukono and Masaka districts, as shown in Table 7.

**Table 7:** Association between occurrence of virulence markers in *E. coli* isolates and diarrhoea status of the piglets

Virulence marker	Category	Diarrhoea status		*p* value
		No	Yes	
STa	Negative	132(80.98)	31(19.02)	0.841
	Positive	10(83.33)	2(16.67)	
STb	Negative	122(81.88)	27(18.12)	0.551
	Positive	20(76.92)	6(23.08)	
EAST1	Negative	98(86.73)	15(13.27)	*0.011
	Positive	44(70.97)	18(29.03)	
F17	Negative	141(81.03)	33(18.97)	0.629
	Positive	1(100)	0(0)	
AIDA-I	Negative	142(81.61)	32(18.39)	*0.037
	Positive	0(0)	1(100)	
F18	Negative	134(81.71)	30(18.29)	0.461
	Positive	8(72.73)	3(27.27)	

## Discussion

This study detected and characterized virulence determinants of *E. coli* associated with the occurrence of diarrhoea in piglets in Ugandan settings. Overall, most of the respondents in the two study districts (75%) were males, which can be explained by the fact that most households in Uganda are headed by males, as also established by Yussif et al. [28]. The present study also revealed that most (92.5%) of the respondents had attained formal education, a key factor in better management practices. Notably, most of the household heads were peasant farmers, which is an indication that agriculture is the key source of income, with a few Ugandans under the formal employment system, while the majority are under small-scale agricultural production. Consistent with the findings of the studies by Ikwap et al. and Aliro et al. [4, 29], most pig farms in Uganda are primarily smallholders, rearing an average of three to five adult pigs, and the majority (> 50%) of the farmers in the study districts owned fewer than 10 pigs. These farmers face a problem of infections, with 63.4% of the respondents in this study reporting encountering a challenge of diarrhoea among the different age groups. Consistent with the findings by Ikwap et al. and Aliro et al. [4, 29] in Northern and Eastern Uganda, diarrhoea was reported by farmers as one of their major challenge. This in turn leads to rampant drug misuse as a way of curbing these infections, a key factor in the development of AMR [15].

When the demographics of the two study districts were compared, Masaka had more respondents who had attained formal education than Mukono, and this difference was statistically significant (*p value* = 0.001). This result could indicate that farmers who are most educated are most likely to have a better understanding of agricultural management principles and practices and hence less animal disease prevalence. This result agrees with a finding stated by Obala and colleagues in their study in Uganda [1]. When infections are minimal, there is minimized and thus more controlled antimicrobial drug use, a key factor in reducing AMR rates.

There was a high prevalence of *E. coli* (81.4%) recovery from faecal samples of piglets from both Masaka and Mukono districts. This result could be attributed to the fact that *E. coli* is a normal gut flora, and thus, its isolation frequency from faecal matter is expected to be high. This finding concurs with that obtained by Kallau et al. [15], who reported a slightly higher (85.4%) prevalence of *E. coli* isolates from pigs. There was also a high isolation frequency of the bacteria from both piglets with (74.4%) and without (83.1%) typical signs of diarrhoea. This could be explained by the fact that *E. coli* is a competitive pathogenic bacterium and thus can still be isolated from a diarrheic piglet sample where gut flora health is expected to be disturbed. Our findings are like those of a previous study by Vidal et al. [30], who also reported no significant difference in the isolation of this bacteria in diarrhoeic and non- diarrhoeic piglets.

Two out of every ten piglets sampled presented with typical signs of diarrhoea, which was probably a trigger for antimicrobial drug administration by farmers and veterinary practitioners. The majority (73.2%) of the farmers reported using antibiotics on their farms, and most of them (95.8%) sought professional veterinary care, which implies that there was prudent use of the antibiotics. However, as noted by Nohrborg et al. [31], selling antibiotics is a source of income to animal health practitioners, and thus, most often, they administer these drugs to obtain some money. Therefore, it was imperative for this study to determine the prevalence of *E. coli* antimicrobial resistance (AMR) against commonly used drugs in pig production. This was based on the fact that *E. coli* is a normal gut flora recommended by the World Organization for Animal Health (WOAH) as an indicator species useful in monitoring and surveillance of AMR [15].

All the *E. coli* isolates from piglets in Masaka and Mukono districts were resistant to erythromycin, as shown in Fig. 1, which could be attributed to misuse of this drug in pig production. The estimates of frequency of antibiotics use are generally unavailable in Uganda. Much as the actual antimicrobial consumption in the farm, at country/district level, are largely unknown, a study by authors in [32], reported 82.8% of the farmers in Wakiso district, Uganda, used antibiotics for treatment of their animals in the previous month with a frequency of use of Erythromycin at 7.8%. This is reflective of high antimicrobial consumption in animal sector, thus, our findings of total resistance to erythromycin call for urgent studies to understand the molecular mechanisms behind this resistance and come up with mitigation measures to combat this great challenge. There was high resistance to tetracycline, ampicillin, and trimethoprim sulfamethoxazole, which can be explained by the fact that these drugs have been reported to be readily available and thus commonly used by farmers in ways that may be imprudent and thus trigger resistance development [7, 15]. There was a high susceptibility of the *E. coli* isolates to gentamicin, chloramphenicol, ciprofloxacin, and nalidixic acid. Chloramphenicol is no longer used in food animal production and thus could explain the high susceptibility levels of the isolates to this drug. Ciprofloxacin and nalidixic acid are also mostly used in human medicine, and thus, the high susceptibility of *E. coli* isolates to the drugs, even though in this case the susceptibility was not up to 100%, may be suggestive of cross resistance and mutation. Gentamicin has been infrequently used in Uganda due to its high cost and thus is unavailable to most farmers [33] however, due to the high resistance against commonly used drugs, farmers have now resorted to the use of this drug, and thus, we see emerging resistance against the drug. Worryingly, the multidrug resistance level found in this study was high (56%), which calls for efforts to mitigate AMR challenge.

The prevalence of *E. coli* virulence gene markers was high, with at least 58.3% of the isolates expressing one of the virulence gene markers screened. Adhesin gene markers (F18, F17 and AIDA-I) were found in 7.5% of the isolates, with fimbrial F18 (6.3%) being the most prevalent adhesin, one isolate expressing F17 and another one having non fimbrial adhesin AIDA-I. More isolate expressed enterotoxin gene markers than adhesins, whereby enterotoxin gene markers (EAST1, STb and STa) were found in 57.1% of the isolates, with EAST1 (35.4%) being the most predominant, followed by STb (14.8%) and STa (6.9%). There was expression of more than one virulence gene marker, and 11 different combinations were obtained, with the enterotoxin STb and EAST1 combination being expressed more frequently. The *E. coli* virulence gene marker prevalence obtained in this study is much higher than that obtained by Obala et al. [1], which was 18.4% in the districts of Central Uganda. A study conducted by Abubakar et al. [7] in South Africa revealed a prevalence of 33%, which is also lower than the 58.3% obtained in this study. This clearly highlights the significance of *E. coli* pathotypes as causers of piglet diarrhoea and mortality in Uganda. The EAST1 gene was the most prevalent (35.4%), as reported by Ikwap et al. [34] and Abubakar et al [7] among South African piglets; thus, there is a need to further understand the precise role of this gene marker possessed by these bacteria in the pathogenesis of enteric colibacillosis. Similar to the results from a study by Ikwap et al. [4], genes encoding fimbriae F5, F6 and F41 were not detected in the current study. However, our study did not reveal any F4 gene marker that was detected by both Obala et al. [1] and Ikwap et al. [4]. The F18 gene was detected in *E. coli* isolates in this study, similar to a previous study performed on commercial pig farms in Central Uganda by Okello et al. [35].

The expression of more than one virulence gene marker is worrying development since it is an indication that the bacterium could evolve into a more virulent pathotype with severe detrimental effects on piglet performance and survival. There was a significant association between the occurrence of diarrhoea in piglets sampled and the presence of EAST1 and AIDA-I virulence gene markers among the *E. coli* isolates from the piglets. This result further highlights the role of the nonfimbrial adhesin AIDA-I in the etiology of piglet diarrhoea [7]. However, the association was nonsignificant for F17, F18, STb and STa, indicating that these genes are expressed by both diarrhoeic and the non-diarrhoeic piglets, which agrees with the findings of Ikwap et al. [4]

In conclusion, this study established a high prevalence of virulence markers among *E. coli* isolates from diarrhoeic piglets; hence, these virulence factors were associated with the cause of diarrhoea in piglets. The level of *E. coli* multidrug resistance against the commonly used antimicrobials in districts of Central Uganda was worryingly high. Many virulent *E. coli* isolates do not have known adhesion genes, and their mechanism of attachment to the host tissue remains obscure. Further studies should be done to elucidate key adhesins employed by this *E. coli* in establishing itself in the intestine of piglets, and this could be used to identify appropriate vaccines for mitigation of colibacillosis in piglets in Uganda. Additionally, urgent measures should be put in place to address rampant AMR.

### Electronic supplementary material

Below is the link to the electronic supplementary material.

Supplementary Material 1

## Data Availability

The datasets generated and analysed during our study are not publicly available as the work is part of an ongoing master’s course but are available from the corresponding author on reasonable request.
