# Supplementary material for: Molecular characterization of Escherichia coli virulence markers in neonatal and postweaning piglets from major pig-producing districts of Uganda
Source: BMC Vet Res. 2024 May 27;20:230. doi: 10.1186/s12917-024-04092-x (PMC11129443; doi:10.1186/s12917-024-04092-x)
Supplement: Supplementary file 1 — Supplementary Material 1 [file 12917_2024_4092_MOESM1_ESM.docx]

**Pig Herd level questionnaire**

The purpose of this questionnaire is to obtain information that can be used to find possible solutions for the prevention / control of colibacillosis in pigs. Please answer the questions honestly and correctly. This is for research purposes and the information given will be kept confidentially.

1. (a) Date…………………………………………………………………………………...

(b) Name of farmer/farm………………………………………………………………...

(c) Farm code …………………………………………………………………………...

(d) Physical address/ location of farm

i) District………………………………………………………………………...

ii) Sub county………………………………………………………………...

ii) Parish ……………………………………………………………………………

iii) Village ………………………………………………………………………….

iv) Latitude…………………………Longitude………………………………….

v) Telephone contact………………………………………………...

**Biodata**

Name of the Head of the House Hold: …………………………………………….

Sex of the Head of the House Hold: Male……. Female…….

Level of education of the Head of the House Hold.

None

Primary

Secondary

Tertiary

Other………………….

Age of Household head (years)

10-20

21-30

31-40

41-50

51-60

> 61

Religion of House hold head

a). Catholic

b). Protestant

c). Muslim

d). Born again Christian

e) Other ……………….

Occupation of the Head of the House Hold.

a). Peasant/farmer

b). Civil servant

c). Self employed

d). Student

e) Other………………………...

Pigs

Who owns the pigs at home?

a). Husband

b). Wife

c). Male children

d). Female children

e) Other ……………………...

Do you keep other animals other than pigs?

Yes

No

If yes, mention the species: (i) Goats……… (ii) Sheep…(iii) cattle……iv) Dogs……v) Other…………...

What breed of pigs do you keep?

Local

Exotic

Both local and exotic

What breeding method do you use?

Own boar

Boar from neighbor

Village boar

What is the size of your pig herd?

1-10

11-20

21-30

>31

How many pigs do you currently have in different age groups?

Piglets 1-7 days old……………………

Piglets 2-4 weeks old…………………...

Pigs 1- 2 months old……………………….

Adult pig >3 months old……………………...

What methods do you use to keep the pigs?

Tethering

Roaming or free ranging

Intensive (pen)

Feeding and Watering of pigs

What do you feed your pigs on?

Roam around

Kitchen leftovers

Commercial feeds

Homemade food

Who usually feeds and takes care of the pigs?

House wife

Husband

Male children

Female children

Hired person

Where do you provide feed and water from?

Feeding troughs

Cemented floor

Non-cemented floor

How often do you clean feeding facilities in no. 16?

Daily

After every two days

Twice a week

Once a week

Do you feed the piglets and adult pigs together?

Yes

No

How is the sow looked after birth?

a). Left to roam around.

b). given kitchen leftovers.

c). given commercial feeds.

d). given homemade feeds.

What is the source of the water used?

River

Well

Bore hole.

Spring

Tap

Rain catchment

When do you always stop the piglets from suckling?

a). 1 week after birth

b). 2 weeks after birth

c). 3 weeks after birth

d). 4 weeks after birth

After stopping piglets from suckling, what do you feed them on?

a). Roam around

b). Kitchen leftovers

c). Commercial feeds

d). Homemade feeds

How many times in a day do you feed the weaned piglets?

a). Once

b). Twice

c). Thrice

d). Continuously

**Pig housing**

From where do the sows usually give birth?

a). In the house

b). Outside the house

c). In the bush

Do you provide housing to neonatal piglets?

a). Yes

b). No

Do you provide housing to weaning piglets?

Yes

No

Do you mix piglets from different litters after weaning?

a). Yes

b). No

How many weaned piglets do you keep per pen?

a). 1-5

b). 6-10

c) 11-15

d) 16-20

e) >21

Does the roof of the pig house leak?

Yes

No

Do you keep piglets and adult pigs together in the same house?

Yes

No

How often do you clean the pig house?

a) Daily

b) After every 2 days

c) Ones a week

d). Occasionally

e). Never

Does the sow and the piglets sleep on the mud?

a). Yes

b). No

**Health management**

Is diarrhea a common condition at your farm?

Yes

No

If yes to 33, which group is commonly / mostly affected?

Neonatal piglets

Weaning piglets

Adult pigs

Are antibiotics commonly used in your farm?

Yes

No

If yes to 35 above, what disease conditions are usually treated with antibiotics?

……………………………………………………………

Do you vaccinate your animals against colibacillosis / piglet diarrhea? Yes……. No………

Do you get professional Veterinary care?

a). Yes

b). No

If yes (in no. 38), how often?

a). Once a month

b). Twice a year

c). Irregularly

e). When pigs are sick

**Pig marketing**

Do you regularly purchase pigs from other farms as replacement stock?

a). Yes

b). No

Who makes decisions on the day to day running of the enterprise?

Husband

Wife

Female children

Male children

Family

Thank you.
